# Enlaces Por La Salud: A Personal Health Navigator Intervention Grounded in the Transnational Framework

**DOI:** 10.1007/s10903-021-01192-w

**Published:** 2021-04-09

**Authors:** Lisa Hightow-Weidman, Joaquin Carcano, Seul Ki Choi, Lynne Sampson, Clare Barrington

**Affiliations:** 1 Institute for Global Health and Infectious Diseases, University of North Carolina, Chapel Hill, USA; 2 Gillings School of Global Public Health, University of North Carolina, Chapel Hill, USA; 3 The Latino Commission on AIDS, New York, USA; 4 School of Nursing, University of Pennsylvania, Philadelphia, USA

**Keywords:** Latinx, HIV, Engagement in care, Intervention transnational

## Abstract

Despite the disproportionate burden of HIV among Latinxs, there is a paucity of culturally appropriate interventions that have shown efficacy at increasing their engagement and retention in HIV care. We describe the development and implementation of Enlaces, a six-session, individual-level intervention, guided by the transnational framework, to improve HIV care outcomes for newly diagnosed and out-of-care Mexican men and transgender women (TW). Descriptive statistics summarizing baseline data and implementation outcomes are provided. 91 participants enrolled between October 2014 and August 2017. Intervention engagement and satisfaction was high; 81.3% completed all six sessions and 100% were very satisfied/satisfied with their experience. Successful implementation of the ENLACES intervention was the result of establishing client trust and maintaining a flexible, supportive approach to intervention delivery. Use of the transnational framework provided a contextualized approach to engaging with Mexican men and TW living with HIV that can be adapted to other Latino populations.

## Introduction

In 2017, Hispanic/Latinx (hereafter, Latinx) made up approximately 10% of North Carolina’s (NC) population [1], a 91% increase since 2004. HIV in NC disproportionately affects Latinx men who have sex with men (MSM) and transgender women (TW). In 2017, eighty-five percent of new HIV cases among Latinx men in NC in 2017 were attributed to male-to-male sex [1]. The rate of HIV infection among Latinx MSM was 830 per 100,000 in 2017, nearly 4 times the rate of White MSM (230 per 100,000) [2]. Among Latinx living with HIV in NC, only 51% were virally suppressed in 2017, the lowest rate of any racial/ethnic group [3]. Further, among MSM with HIV in NC, 61% of Latinx MSM are virally suppressed compared to 70% of white MSM [2]. In 2017, a total of 86 transgender people were estimated to be living with HIV in NC, 15% of whom are Latinx. This is likely an underestimate given that transgender identity was not routinely captured in the NC surveillance system until 2015, limiting accurate surveillance and measurement [1].

Latinx MSM and TW’s access to and engagement in HIV care is shaped by the interplay between factors including immigration and labor policies, intersecting stigma and discrimination, social isolation, and limited access to culturally competent health services [4–8]. Undocumented Latinx immigrants are less likely to have a usual source of care than those who are documented and concerns about job loss for requesting time off as well as fear of deportation from service utilization limits engagement and leads to poor outcomes [9–11].

Qualitative studies conducted with Latinx men and TW in NC underscore the interplay of the migration, HIV vulnerability and the HIV care experiences of this population [12, 13]. Key findings from the formative research conducted to inform the development of the intervention described in the current study include the salience of the mental health burden of diagnosis and the importance of participants’ social networks both within the US and country of origin for provision of support [14]. Most participants had experienced interruptions in their care due to both intersecting stigmatized identities (e.g. being gay, Latinx, undocumented) and intersecting structures (healthcare, immigration policy, institutionalized homophobia). Undocumented participants directly connected their immigration status to their ability to get work, which then affected their retention in HIV care and treatment adherence [14]. Participants also describe using a range of strategies to exert control over their lives with HIV including: understanding their infection, engaging with health care, and developing relationships with health professionals [15].

Despite recognition of the disproportionate burden of HIV among Latinxs, there is a paucity of culturally appropriate interventions that have shown efficacy at increasing engagement and retention in care for Latinx living with HIV especially for those who identify as sexual or gender minorities [16]. Project STYLE (Strength Through Youth Livin’ Empowered), an intervention for Black and Latinx young men who have sex with men living with HIV which included a social marketing campaign targeting youth and members of their sexual and social networks, testing and community outreach, and a tightly linked medical–social support network resulted in increased HIV diagnoses, timely engagement in care and improvements in overall retention rates. However, only 11% (n = 9) of the study sample identified as Latinx [17]. Anti-Retroviral Treatment and Access to Services (ARTAS), an individual-level, multi-session, intervention based on the Strengths-based Case Management (SBCM) model, was found to significantly increase engagement in care at both 6 and 12 months [18]. While the ARTAS intervention was not developed specifically for Latinx populations, in multivariate analysis, the intervention was significantly (p < 0.05) stronger among Latinx participants than other racial/ethnic groups combined [19].

Effective strategies to support engagement in care, adherence to antiretroviral therapy (ART) and viral suppression are needed in new Latinx settlement states, such as NC. To this end, we developed Enlaces por la Salud (hereafter, Enlaces) to improve HIV continuum of care outcomes (e.g. retention in HIV care, viral suppression) for newly diagnosed and out-of-care Mexican men and TW. In this paper, we describe the development and implementation of Enlaces; baseline characteristics of the study sample are provided.

## Methods

### Intervention Overview

We developed Enlaces as part of a 5-year Special Project of National Significance (SPNS) funded by the Health Resources and Services Administration (HRSA). All HRSA grantees developed innovative HIV linkage and engagement program using a transnational approach tailored to either Mexican or Puerto Rican participants. Further, interventions were specifically developed to focus on populations who were newly diagnosed within the past six months or out-of-care (diagnosed with HIV more than six-months ago with gaps in care > 6 months). While both US and foreign-born Latinx populations in NC represent a diversity of cultural/ethnic origins, the majority of Latinx residents identify Mexican as their primary ancestry [20], thus, Mexico was chosen as the country of origin upon which Enlaces was developed. Development of Enlaces was interdisciplinary, including clinical and public health researchers, community advocates, peer navigators and case managers.

### Theoretical Underpinnings

Enlaces was informed by concepts related to transnationalism, migration and stigma. Transnationalism refers to networks, resources and experiences in both countries of origin and countries of settlement and connections between the two [21]. Transnationalism is enacted via communication, social and economic exchanges, and travel which allow individuals to be connected, engaged, and influenced by two or more communities simultaneously [21]. We also drew upon Zimmerman, Kiss, and Hossain’s Migratory Process Framework (MPF) [22], a rights-based model that considers migration to be cyclical and multi-staged, dividing the process into five phases: (1) Pre-departure; (2) Travel; (3) Destination; (4) Interception; and (5) Return. Each of the phases involves distinct health-related risk exposures with cumulative effects over the course of migration, and each also presents distinct opportunities for intervention. The MPF recognizes the transnational experience of migrants across all stages of the framework. In our intervention sessions, we applied the MPF as we considered how stages of migration and migration experiences may influence engagement with HIV care (Table 1). We also applied Parker and Aggleton’s conceptualization of HIV-related stigma as the product of multiple, overlapping forms of stigma related to sexuality, gender, class, and race/ethnicity to the intervention content (Table 1) [23].

**Table 1:** Enlaces session description

Session number	Session name	Main topics	Transnational goal	Migratory processes framework
1	Life and migration history	Life prior to migration, reason for migrating, life in North Carolina, Connection to friends/family in Mexico	Migration history and identification of relevant events or experiences (highlighting strengths) that may shape the HIV care and treatment experience	Pre-departure, travel, destination, interception
2	Healthcare and medical visits	Health history timeline, previous healthcare providers/experiences, health beliefs and practices, differences in care between US and Mexico	Healthcare history prior to, during, and following migration to provide context for initiation or re-engagement with care	Pre-departure, travel, destination
3	Social networks	Social support, network inventory of meaningful relationships in clients’ life both in US and Mexico, cultural issues within social networks	To elicit a social network and support inventory (both local and transnational) to understand the social context in which the client currently lives. To identify messages surrounding HIV status clients are receiving from their community and how this affects them	Destination
4	Stigma and discrimination	Experiences involving stigma in US and Mexico, coping with HIV with support from different social networks	To identify individuals in their social support networks who they would like to disclose their status to and practice the language they want to use in talking about their HIV infection	Destination
5	Healthy living	Cultural beliefs and practices around health (nutrition, exercise, mental health, substance use), experiences with medication in Mexico and US, adherence strategies	To identify the client’s responsibilities as a migrant to improve the understanding of external pressures that may impact healthy living, HIV care and treatment behaviors and outcomes	Destination
6	Self-management and transition plan	Social networks and impact on continued engagement in care, balancing health, work, life priorities as it relates to migration and connection to Mexico	Define future plans with regard to migration and relationships with country of origin and North Carolina	Return

Migratory processes framework stages: (1) Pre- departure, which includes the social, behavioral, and environmental factors affecting migrants before they leave their place of origin; (2) Travel, which addresses the experiences of migrants in transit between their social, behavioral, and environmental factors affecting migrants before they leave their place of origin and intended destination; (3) Destination, with a focus on the conditions of temporary or long- term settlement in a new location; (4) Interception, when applicable, refers to what happens during time spent in detention by immigration authorities; and (5) Return, which focuses on issues faced when migrants go back to their place of origin temporarily or permanently

The core elements of the ARTAS strengths-based case management model informed intervention format (e.g., structured individual sessions with each client) and delivery (e.g., maintaining a client-driven approach and encouraging them to identify and use strengths, abilities and skills to engage in medical care) [18]. Adaptations were made to: (1) ensure all materials were both culturally and linguistically appropriate and acknowledge the transnational perspective; (2) include content relevant for those newly diagnosed as well as those who had fallen out of care; and (3) extend intervention content and duration (from 90 days to 6 months) to address long-term retention and viral suppression.

### Intervention description

Enlaces included six-sessions delivered to individuals over six months by two bilingual Personal Health Navigators (PHNs) located in the Raleigh-Durham-Chapel Hill and Charlotte metro areas of NC. Each session was anchored in the transnational framework, starting with participant’s migration story, and addressed their experiences with health care and stigma, language preferences, and social networks in both NC and Mexico. Each session had a transnational goal that guided the content and activities. PHNs employed a strengths-based perspective, to provide support, help participants identify individual resources and address previous or anticipated barriers to care and promote long-term retention [24].

Session descriptions. And transnational goals are provided in Table 1. Engagement in the program and Session 1 began with the participant sharing their life story pre and post-migration to the US, if applicable, to operationalize of the transnational approach. This allowed the PHN to have a comprehensive overview of the participant’s history and the impact on their current daily life, particularly healthcare management, which laid the groundwork for the ongoing relationship. Important questions included: (1) How are participants still connected to their country of origin?; (2) How does their migration story continue to affect their current life situation?; (3) What has been their experience in the US? Content covered throughout the course of the intervention included HIV diagnosis, appointment debriefs, general HIV knowledge, and working with healthcare teams (Session 2), social networks and support (Session 3), stigma and disclosure (Session 4), health history, healthy living, mental health, and medication adherence (Session 5), and transition plans post-Enlaces (Session 6).

PHNs were required to demonstrate both Spanish and English proficiency and have experience in delivering case management. PHNs underwent multiple Enlaces training, including an initial webinar by The Latino Commission on AIDS. The webinar provided project background, relevant research findings, and an introduction to HIV care provision. This was followed by a 2 day in-person training which focused on the structure of strengths-based case management, how to interact with participants and people of diverse backgrounds, and, reviewing paperwork and the process involved in connecting a participant to social services. Other highlights included demonstrations and role-plays of the role and responsibilities of the PHN, recognizing the principles of a participant -centered, goal setting, and establishing effective community collaborations and relationships for seamless linkage and referrals to care.

### Study sample

Participants were referred to Enlaces between October 2014 through August 2017 from health care providers and staff at HIV care clinics, the state’s disease intervention specialists (DIS) and state bridge counselors (SBC) who work with newly diagnosed and out-of-care individuals respectively, area HIV service organizations, and by running periodic (e.g. bimonthly) clinic out-of-care lists and providing these referrals to PHNs. Eligibility criteria were: (1) self-identifying as Mexican/Mexican–American; (2) Male gender assigned at birth; (3) Diagnosed HIV-positive; (4) 18 years or older; (5) Able to provide informed consent; and (6) English or Spanish speaking. Participants had to either be newly diagnosed (within the past 6 months) or out-of-care (diagnosed with HIV more than 6-months ago and not being seen regularly by an HIV primary care provider (e.g. gaps in care > 6 months). Frequent cancellations and rescheduled appointments as well as use of walk-in clinic hours were used as indicators of inconsistent engagement in care, as determined by a medical chart review. Ten referred clients did not qualify for the study (did not identify as Mexican/Mexican American, not newly diagnosed or out-of-care). Of the remaining 110 participants who were eligible, eight did not attend the initial enrollment visit and 11 either refused to participate or were unable to be contacted, for a total of 91 enrolled participants. Assuming that the prescreened pool was all eligible (n = 110), this study achieved 83% cooperation.

The study was approved by the Institutional Review Board (IRB) at the University of North Carolina (IRB #13-3673).

### Data Collection

Upon confirmation of eligibility, participants provided informed consent, which included a Health Insurance Portability and Accountability Act (HIPAA) waiver for release of their medical information related to HIV clinical care. Participants completed surveys at baseline, 6 months and 12 months. Surveys lasted approximately 1 − 1 ½ hours and participants received $30 compensation for completion of each survey. A comprehensive quantitative assessment was comprised of measures that were pulled from the literature and when possible, validated and/or published in Spanish. All items in English were translated by a professional Spanish document translator. The full list of items was then reviewed by a team of two native Spanish speakers. Discrepancies were resolved iteratively, including pilot testing the measures with native Spanish speakers at study sites. Surveys were administered using a computer assisted survey instrument in the participants’ language of choice (English or Spanish). Online data from the baseline survey and intervention satisfaction questionnaire were used for this analysis. All data were stored in a secure server maintained by the HRSA Evaluation and Technical Assistance Center.

### Measures

#### Demographic and Psychosocial Variables

Demographic variables included age, gender identity, education, income and relationship status.

Alcohol Use was assessed using the Alcohol Use Disorders Identification Test (AUDIT-C) [25, 26], scored on a scale of 0–12, with 0 reflecting no alcohol use. In men, a score of 4 or more is considered positive for a substance use disorder. Depression was assessed with the CES-D using 10 questions scored from 0 to 3 (0 = rarely or never to 3 = most or all of the time). Total scores range from 0 to 30 [27]. Higher scores suggest greater severity of symptoms with scores ≥ 10 = depressive symptoms.

#### Transnational Practices, Migration History and Acculturation

Individual items measured country of origin (COO), years in the US, and first language spoken. Ties to participants COO was assessed by questions that measured how often they travel to, send money or goods to their COO.

The Bidimensional Acculturation Scale, consisting of three subscales (Language use, Linguistic proficiency and Electronic media) was also used [28]. Answers to 12 items that measure each cultural domain (Latinx and US) were averaged across items for each respondent. A sample item from Language use is: How often do you speak English/Spanish with your friends? A sample item form Linguistic proficiency is How well do you read English/Spanish? A sample item from Electronic media is How often do you watch television programs in English/Spanish? Each respondent was assigned two scores: (a) one for the average of the 12 items making up the Latinx domain and (b) another score for the 12 items forming the US domain. The possible total score range is from 1 to 4 for each cultural domain. Based on the literature, a score of 2.5 was used as a cutoff score to indicate low or high level of adherence to each cultural domain.

The US cultural identity subscale was based on six items from the American Identity Questionnaire [29]. A parallel six-item subscale was developed to assess Latinx identity as part of the Abbreviated Multidimensional Acculturation Scale (AMAS–ZABB) [30]. Thus, two separate scores are obtained for identity: one for US-American identity and one for Latinx identity. Sample items include: I think of myself as US-American (or Latinx) and being US American (or Latinx) plays an important part in my life. Each were scored on a 4-point Likert scale (1 = strongly disagree, 4 = strongly agree) with higher scores indicating greater identification with culture of origin.

#### Intervention Satisfaction

Participants completed a paper-based questionnaire at 12 months to assess intervention satisfaction. Four questions, rated on a four-point Likert scale from very satisfied to very dissatisfied included “How satisfied are you with your experience participating in the ENLACES intervention?” “How satisfied are you with the services, if any, you were linked to during ENLACES?” “How satisfied are you with the skills you learned and/or enhanced by participating in the intervention?” “How satisfied are you with the Personal Health Navigator you worked with over the course of the intervention?” One question, “Would you recommend ENLACES to anyone you know?” had a dichotomous outcome (yes/no).

### Statistical Analysis

Univariate and bivariate analyses were conducted to describe the population at baseline, based on the categorization of new to care or re-engaging. All data was analyzed using SAS 9.4 (SAS Institute; Cary, North Carolina).

## Results

### Sample Demographics

Baseline characteristics of Enlaces participants by HIV care status is provided in Table 2; 49 were new to care and 42 re-engaging. The majority identified as male (89.9%). Mean age was 36.8 years and the majority identified as male (89.9%). Participants reported substantial socioeconomic barriers including high levels of poverty (60.5% had a total household income of < $11,490 in the prior year), past incarceration (33.0%) and lack of healthcare coverage (81.3%). Approximately one-quarter reported being unstably housed in the 6 months prior to study entry. Approximately one-third (30.8%) of the sample screened positive for an alcohol use disorder and 18.7% reported use of other substances (non-injection). Compared to participants re-engaging in care, those new to care were about 5 years younger (34.6 years vs. 39.4 years; p = 0.04). There were no other statistically significant differences between the two populations.

**Table 2:** Baseline characteristics of enlaces participants by HIV care status

Variable	Total sample (n = 91)	New to care (n = 49)	Re-engaging (n = 42)	p-value
Age, mean [SD]	36.8 [10.9]	34.6 [10.5]	39.4 [11.0]	0.04
Gender preferred (n = 79)				0.83
Male	71 (89.9%)	39 (88.6%)	32 (91.4%)	
Female	2 (2.5%)	1 (2.3%)	1 (2.9%)	
Trans-female or transgender	2 (2.5%)	2 (4.6%)	0 (0%)	
Other	4 (5.1%)	2 (4.6%)	2 (5.7%)	
Highest education				0.78
Up to 8th grade	33 (36.3%)	20 (40.8%)	13 (30.9%)	
9th–11th grade	25 (27.5%)	13 (26.5%)	12 28.6%)	
Grade 12 or GED	16 (17.6%)	8 (16.3%)	8 (19.1%)	
Some college or above	17 (18.7%)	8 (16.3%)	9 (21.4%)	
Relationship status				0.66
Single	58 (63.7%)	30 (61.2%)	28 (66.7%)	
In a relationship	33 (36.3%)	19 (38.8%)	14 (33.3%)	
Annual household income				0.95
≤ $11,490	55 (60.5%)	31 (63.3%)	24 (57.1%)	
> $11,490	36 (39.5%)	18 (36.7%)	18 (42.9%)	
Ran out of money in ≥ 3 months out of past 6	24 (26.4%)	11 (22.4%)	13 (30.9%)	0.31
Had to borrow money^a^	61 (67.0%)	35 (71.4%)	26 (61.9%)	0.38
Sometimes/often hungry^a^	14 (15.4%)	5 (10.2%)	9 (21.4%)	0.35
Unstable housing^a^				0.11
Rarely/sometimes	19 (20.9%)	11 (22.4%)	8 (19.1%)	
Often	4 (4.4%)	0 (0%)	4 (9.5%)	
Jail/prison (lifetime)	30 (33.0%)	17 (34.7%)	13 (30.9%)	0.91
Jail/prison^a^	11 (36.7%)	6 (35.3%)	5 (38.5%)	1.00
Gender identity of sex partners^a^				
Male	43 (47.2%)	27 (55.1%)	16 (38.1%)	0.14
Female	22 (24.2%)	10 (20.4%)	12 (28.6%)	0.46
Genderqueer	1 (1.1%)	0 (0%)	1 (2.4%)	0.46
Not had sex	19 (20.9%)	10 (20.4%)	9 (21.4%)	1.00
No health coverage	74 (81.3%)	43 (87.8%)	31 (73.8%)	0.11
Proportion with alcohol disorder (AUDIT-C ≥ 8)	28 (30.8%)	16 (32.6%)	12 (28.6%)	0.82
Non-injection drug use^1^	17 (18.7%)	11 (22.5%)	6 (14.3%)	0.66
Depressed (CES-D 10 ≥ 10)	36 (40%)	21 (43.8%)	15 (35.7%)	0.52

^a^Time frame is past 6 months

### Transnational Practices

Migration history, transnational practices and acculturation are described in Table 3. Most participants were born in Mexico (87.8%), spoke Spanish as their primary language (94.4%) and immigrated to the US at a mean age of 20.1 years. While participants rarely traveled back to Mexico, most occasionally or regularly sent money (70%) or goods (45.5%). Mean scores were higher on the Latinx scales compared to the US Scales, indicating greater identification with Latinx culture and language on both the Bidimensional Acculturation Scale (3.48 vs. 2.36) and cultural identity scales (3.81 vs. 2.30). There were no significant differences related to transnational practices between new to care and re-engaging participants.

**Table 3:** Transnational practices among enlaces participants

Variable	Total sample (n = 91)	New to care (n = 49)	Re-engaging (n = 42)	p-value
Country born				1.00
US	9 (10.0%)	5 (10.4%)	4 (9.5%)	
Mexico	79 (87.8%)	41 (85.4%)	38 (90.5%)	
Other	2 (2.2%)	2 (4.2%)	0 (0%)	
Arrival in the United States (N = 80)				0.15
Mean [SD]	20.1 [8.8]	18.8 [8.1]	21.7 [9.4]	
First spoken language				0.51
English	3 (3.3%)	1 (2.1%)	2 (4.8%)	
Spanish	85 (94.4%)	45 (93.8%)	40 (95.2%)	
Both	2 (2.2%)	2 (4.2%)	0 (0%)	
Bidimensional acculturation scale				
US: mean [SD]	2.36 [0.85]	2.37 [0.89]	2.36 [0.79]	0.96
Latinx: mean [SD]	3.48 [0.56]	3.47 [0.61]	3.49 [0.51	0.89
Cultural identity (US)				
US: mean [SD]	2.30 [0.95]	2.42 [0.99]	2.17 [0.90]	0.22
Latinx: mean [SD]	3.81 [0.46]	3.84 [0.39]	3.78 [0.53]	0.56
How often travel to country of origin (COO)				0.95
Never/rarely	79 (87.8%)	43 (89.6%)	36 (85.7%)	
Sometimes/often	11 (12.2%)	5 (10.4%)	6 (14.3%)	
Send money to COO				0.09
No	27 (30.0%)	18 (37.5%)	9 (21.4%)	
Yes, occasional	45 (50.0%)	24 (50.0%)	21 (50.0%)	
Yes, regularly	18 (20.0%)	6 (12.5%)	12 (28.6%)	
Send goods to COO				0.50
No	49 (54.4%)	29 (60.4%)	20 (47.6%)	
Yes, occasional	29 (32.2%)	13 (27.1%)	16 (38.1%)	
Yes, regularly	12 (13.3%)	6 (12.5%)	6 (14.3%)	
Contact with those in COO				0.36
Mean [SD]	2.49 [0.94]	2.41 [0.95]	2.59 [0.92]	

### Implementation Outcomes and Satisfaction

Overall sample retention was 78.0% (n = 71) at 6 months and 79.1% (n = 72) at 12 months. The majority of participants (n = 74, 81.3%) completed all six sessions. A third of the sample (n = 33, 36%) received more than one session in a single encounter, with 23% of all encounters involving multiple sessions. The mean number of days from the first session to the last was 166 days (~ 5 and a half months). Overall satisfaction with the intervention was high (n = 72); 100% were very satisfied/satisfied with their experience in the intervention, 98.6% were very satisfied/satisfied with the services they were linked to during ENLACES, 98.6% were very satisfied/satisfied with the skills learned and/or enhanced by participating in the intervention, and 100% were very satisfied/satisfied with the PHN. All participants reported being willing to recommend Enlaces to others in need of similar services.

## Discussion

This paper describes the foundation and development of Enlaces, a HRSA-supported SPNS intervention designed to improve HIV outcomes among Mexican men and TW. Given that HIV rates continue to rise among Latinx populations in the Southern US, novel interventions to facilitate diagnosis, entry and retention in care are urgently needed. Enlaces is, to our knowledge, the first transnational intervention developed to address barriers that Latinx men and TW face along the HIV care continuum, including stigma, cultural beliefs and practices around health and social networks of support.

Enlaces was developed through a strengths-based perspective; which acknowledged the individual and structural barriers to care faced by participants and prioritized their personal strengths and resiliency to address them. Indeed, at baseline, participants reported experiencing substantial socioeconomic barriers to navigating medical care including high rates of financial insecurity (e.g., low annual incomes, housing and food insecurity) along with notably high rates of drug and alcohol use. Provision of tailored strengths-based solutions including an assessment and understanding of transnational practices may help to assist participants in successfully navigating these challenges.

Transnationalism was evident among participants, most of whom who maintained strong social, cultural and economic ties with their COO despite living in the US for many years (mean 20.1 years) and traveling back to Mexico rarely or not at all [31, 32]. Higher scores on the Bidimensional Acculturation Scale and cultural identity measure indicate that participants tended to have greater identification with their Latinx ethnic identity, living their lives and communicating mostly in Spanish. Consideration of these ties and the spaces they occupy within the daily lives of migrants is crucial for understanding the complex role that transnational networks can play in both promoting and hindering engagement in healthcare and self-management of chronic health conditions [32, 33]. The impact of transnationalism on the health behaviors of Latinx populations living with HIV has not been fully elucidated; a gap that this project seeks to fill through both quantitative and qualitative analyses that are forthcoming.

We found overall high levels of engagement with both the intervention as well as retention in the study. This may be attributed to our use of the transnational framework, which encouraged a holistic approach and facilitated trust between PHNs and the clients. In addition, PHNs engaged in frequent contact with clients through phone calls and text messages and maintained flexibility with regard to scheduling sessions (e.g., completing multiple sessions during an encounter, conducting sessions during weekends or evening hours). This was critical given that many participants had issues with transportation and maintained work schedules that did not allow time off, including jobs that required them to spent long spans of time working out-of-state.

Future interventions with Latinx populations should consider similar strategies to encourage participation.

While we faced initial difficulties in building referral networks, gaining buy-in from providers and clinics and establishing our program's presence and reputation, we leveraged team member’s long-standing history and local trust of our community partners and engaged in visibility campaigns (through radio shows, attending regional conferences, presentations to providers, and numerous meetings with area staff) to inform them of our services and outline mutually beneficial strategies of referrals and care engagement.

### Limitations

While the primary focus of the Enlaces intervention was on Mexican MSM and TW, a decision to enroll all participants born male was made given prior research that found that immigrant sexual minority Latinx men may be reluctant to disclose their sexual orientation [34]. The focus on the subpopulation of Mexicans living in NC may limit the generalizability of our findings given that their HIV related healthcare experiences and barriers and facilitators to care may differ from Latinx populations currently residing in other US states or with different ancestries. Finally, while we do not ask about immigration status, within Enlaces sessions, PHNs explicitly address how fears of deportation, as well as time and economic pressures may exacerbate barriers to care [35, 36].

## New Contribution to the Literature

Enlaces is a novel intervention developed to improve HIV continuum of care outcomes for newly diagnosed and out-of-care/inconsistently-in-care Mexican men and TW. Future analyses will determine the effectiveness of Enlaces at increasing engagement in care and viral suppression. Increasing the proportion of Latinx individuals living with HIV who are engaged in care and virally suppressed will help to achieve the national goals of reducing new infections and decreasing health disparities; thus, moving closer towards the ultimate goal of ending the epidemic [37].
